# Supplementary material for: Mechanisms governing the pioneering and redistribution capabilities of the non-classical pioneer PU.1
Source: Nat Commun. 2020 Jan 21;11:402. doi: 10.1038/s41467-019-13960-2 (PMC6972792; doi:10.1038/s41467-019-13960-2)
Supplement: Supplementary file 7 — Source data [file 41467_2019_13960_MOESM7_ESM.zip › Source_Data/Figure6/Figure6B_MotifScanOutput/FLI1_mutPU.1/homerResults/motif18.similar.html]

motif18

## Information for motif18

C
T
A
G
G
A
T
C
C
G
A
T
C
T
A
G
G
C
A
T
A
G
T
C
C
G
T
A
A
C
T
G
  
Reverse Opposite:  

T
G
A
C
G
C
A
T
T
C
A
G
C
G
T
A
G
A
T
C
G
C
T
A
C
T
A
G
G
A
T
C
  

|  |  |
| --- | --- |
| p-value: | 1e-67 |
| log p-value: | -1.550e+02 |
| Information Content per bp: | 1.601 |
| Number of Target Sequences with motif | 3462.0 |
| Percentage of Target Sequences with motif | 33.74% |
| Number of Background Sequences with motif | 9536.5 |
| Percentage of Background Sequences with motif | 25.02% |
| Average Position of motif in Targets | 97.9 +/- 56.7bp |
| Average Position of motif in Background | 100.2 +/- 62.3bp |
| Strand Bias (log2 ratio + to - strand density) | -0.0 |
| Multiplicity (# of sites on avg that occur together) | 1.20 |
| Motif File: | file (matrix) reverse opposite |

### Similar de novo motifs found

|  |  |  |  |  |  |  |  |
| --- | --- | --- | --- | --- | --- | --- | --- |
| Rank | Match Score | Redundant Motif | P-value | log P-value | % of Targets | % of Background | Motif file |
| 1 | 0.813 | T G C A C G A T T A G C C G A T C T A G C A G T A T G C C T G A A C T G | 1e-47 | -110.186589 | 8.87% | 4.89% | motif file (matrix) |
| 2 | 0.808 | T G C A A C G T C T A G C G T A G T A C C T G A A C T G | 1e-37 | -86.207956 | 18.77% | 13.58% | motif file (matrix) |
